# Prevalence of and factors associated with the use of methylphenidate for cognitive enhancement among university students

**DOI:** 10.31744/einstein_journal/2020AO4745

**Published:** 2019-10-17

**Authors:** Raissa Carolina Fonseca Cândido, Edson Perini, Cristiane Menezes de Pádua, Daniela Rezende Junqueira

**Affiliations:** 1 Universidade Federal de Minas Gerais, Belo Horizonte, MG, Brazil.

**Keywords:** Methylphenidate, Cognition/drug effects, Central nervous system stimulants, Off-label use, Students, Prevalence

## Abstract

**Objective:**

To estimate the prevalence of and factors associated with the use of methylphenidate for cognitive enhancement among undergraduate students.

**Methods:**

Simple random sample of students of the *Universidade Federal de Minas Gerais* (n=438), invited to answer an online questionnaire about the use of methylphenidate. Data collection occurred from September 2014 to January 2015. The sample was described by means of proportions, means and standard deviations. A multivariate analysis was performed using the Classification and Regression Tree algorithm to classify the cases of use of methylphenidate for cognitive enhancement in groups, based on the exposure variables.

**Results:**

Out of 378 students included, 5.8% (n=22) reported using methylphenidate for cognitive enhancement; in that, 41% (9/22) in the 4 weeks prior to the survey. The housing situation was the variable most often associated with the use of methylphenidate for cognitive enhancement. Eleven students reported using methylphenidate for cognitive enhancement and other purposes 4 weeks prior to the survey, 27% of whom had no medical prescription to purchase it.

**Conclusion:**

The use of methylphenidate for cognitive enhancement is frequent among Brazilian undergraduate students and should be considered a serious public health problem, especially due to risks of harm and adverse effects associated with its use.

## INTRODUCTION

Methylphenidate is a central nervous system stimulant approved in Brazil for the treatment of Attention *Deficit* Hyperactivity Disorder (ADHD) and narcolepsy. The use of psychostimulant substances for cognitive enhancement, such as methylphenidate, extended the practice of doping beyond the sports environment. In many countries, university students have been using medications to improve their performance in assessments, and enhance their learning ability, creating a parallel market for substances on university campi.^1 , 2^ The use of methylphenidate for pharmacological cognitive enhancement is highlighted in this context, with an estimated prevalence of 5 to 7% among American undergraduate students.^3 - 5^ The Brazilian regulation lists methylphenidate as a psychotropic drug, along with other substances that induce addiction, such as methamphetamine (“ice”), and their prescription and use are controlled by special notification requirements.^6 , 7^

Most analyses on consumption of methylphenidate for non-medical purposes deal with issues related to drug abuse and addiction.^3^ Few studies investigate the prevalence of the use of stimulants for non-medical purposes, analyzing comprehensively the behavior of individuals who report the use of these drugs for cognitive enhancement.^3 - 5^ Scientific evidence of the efficacy and adverse effects of methylphenidate in cognitive enhancement is also scarce.^8^ However, the abusive use of psychostimulants is considered a public health problem in the United States and the United Kingdom, where the research and debate around the subject are intensifying.^9 , 10^

In Brazil, a study on the social representation of the use of methylphenidate and pharmacological cognitive improvement in a group of university students evidenced this practice as a reality in our country.^11^ Considering this reality and the lack of information on this context, it is relevant to evaluate the prevalence of the consumption of methylphenidate for cognitive enhancement in university settings.

## OBJECTIVE

To determine the prevalence of and the factors associated with the use of methylphenidate by Brazilian university students for purposes of cognitive enhancement.

## METHODS

### Study design

This was a descriptive cross-sectional study with university students from the *Universidade Federal de Minas Gerais* (UFMG). Headquartered in the city of Belo Horizonte, UFMG is one of the largest universities in the country, with 47,088 students enrolled in the year 2014, when data collection was performed.^12^

### Participants

The students were selected from the university’s registration data. To assess the representativeness of the resulting sample, the students were divided into groups: three groups corresponding to the major areas of knowledge (Exact sciences, Human sciences, and Biological/Health sciences) for undergraduate students, and another group, called graduation group, for graduate students and medical and multidisciplinary residency students. The students’ field of knowledge was included in the UFMG register and was also informed by the participants in the research questionnaire.

### Data collection

The registration information of all students enrolled in the UFMG was organized in a database, and a code was assigned for each student. A simple random sample of students was selected by lot and recruited by electronic mail. The students received three invitations at 15-day intervals. Non respondents were systematically replaced to provide for the construction of a simple random sampling without replacement. Non respondents after the third contact, once all replacements have been exhausted, were considered losses.

Data were collected using an electronic questionnaire, organized in three parts: sociodemographic variables; data on methylphenidate consumption; and data on lifestyle habits, including physical activity practice, smoking habit and illicit drug use. The practice of physical activity was analyzed in four categories: no physical activity (reference); low frequency (30 to 45 minutes, three times per week); medium frequency (30 minutes, five times per week, total of 2 hours and 30 minutes per week); and high frequency (more than 2 hours and 30 minutes per week). The use of methylphenidate in the 4-week period prior to the survey was classified as “recent”. The report of use of methylphenidate over a period of 4 weeks corresponded to use at any time in life.

For the collection and storage of data, we used the SurveyMonkey^®^ electronic platform.

### Sample calculation

Considering a prevalence of methylphenidate consumption for cognitive enhancement of 7%,^4^ a sampling error of 4%, a two-tailed significance level of 5%, and a power of 80% for the study, adopting a 25% loss rate, we calculated a simple random sample of 438 students enrolled in UFMG undergraduate, graduate and residency courses (calculated sample of 350, disregarding the loss rate).

### Statistical analysis

The response records were manually reviewed for errors and inconsistencies. The analyzes were performed using the IBM software (SPSS) Statistics Base, version 22.0. Dichotomous variables were described using proportions; continuous variables were described using mean and standard deviation. The analysis of the representativeness of the sample in relation to the population according to the area of knowledge was performed using the *z* test. The prevalence of methylphenidate consumption was calculated with a 95% confidence interval (95% CI).

To understand the use of methylphenidate for cognitive enhancement in relation to the exposure variables, a multivariate analysis was performed using the decision tree method. By means of the Classification and Regression Tree (C&RT) algorithm,^13^ the decision tree method was used to investigate predictors of methylphenidate consumption for cognitive enhancement as an alternative to the binary logistic regression method, given the limited number of cases available for analysis.

The decision tree is a recursive partitioning technique used for data classification and regression in smaller strata, defining subsets (child nodes) as homogeneous as possible in relation to the root node (total population containing the dependent variable). The C&RT method maximizes node homogeneity, and the extent of case heterogeneity is an indication of impurity. The measure of impurity used was the Gini coefficient, which is based on the squared probabilities of association for each category of the dependent variable. The Gini coefficient varies between zero and 1, and reaches its minimum value when all cases in a node are included in the same category (pure node). The difference between the Gini coefficient for the root node and the sum of the values for the child nodes, weighted by the proportion of cases in each child node, is presented in the tree as improvement. This measure represents the relative importance of the node, evaluated by the decrease in the heterogeneity of the branch, when compared to the root node (total population). The adjustment of the final model was assessed by the risk estimate, which identifies the proportion of cases that were incorrectly classified by the model.^14^ Exposure variables for the medication use were included simultaneously in the model, and those with higher improvement values remained in the final model.

The study was approved by the UFMG Research Ethics Committee (CEP 441,603), CAAE: 19606913.5.0000.5149.

## RESULTS

A total of 378 students were included, of which 69% were in undergraduate courses. The sample was similar to the university population for area of knowledge, despite the higher concentration of respondents in the areas of Biological and Health Sciences and Human Sciences ( Table 1 ).

**Table 1:** Distribution of students’ characteriscts in the study sample and in the university population by area of knowledge

Area of knowledge	Sample (n=378)	Study university (n=47,088)
Undergraduate courses	105 (27.8)	9,367 (20)*
Biological/Health sciences	65 (17.2)	10,053 (21)
Physical sciences	90 (23.8)	14,453 (31)^†^
Human sciences	118 (31.2)	13,215 (28)
Graduate, Medical and Multidisciplinary Residency Courses	105 (27.8)	9,367 (20)*

Results expressed as n (%). * p=0.0002 (z test); ^†^ p=0.0026 (z test).

The mean age of students was 27.9 years (standard deviation of 8.2), and women accounted for the majority of the sample (64%). Most participants reported being single and living with their parents, and the predominant monthly family income range was 3.1 to 5 minimum wages (30.2%). A significant portion of the students did not practice physical activity regularly (45%). Most interviewees reported using alcohol (55%) and declared being non-smokers (91.5%). Consumption of medications other than methylphenidate was reported by 34% of students. The use of illicit drugs and opioid drugs was declared by 39% of sample, occurring mostly for 12 months or more (40% and 44%, respectively) ( Table 2 ).

**Table 2:** Demographic and socioeconomic characteristics, habits and lifestyle of students

Variables	
Sex	
Female	241 (63.8)
Age, years	
≤19	5 (1.3)
20-30	285 (75.4)
>30	88 (23.3)
Marital status	
Single	289 (76.4)
Married	62 (16.4)
Others*	27 (7.2)
Living arrangement^†^	
With parents	183 (48.5)
With partner	73 (19.4)
With other family members	35 (9.3)
With friends	18 (4.8)
Student housing	45 (11.4)
On their own	23 (6.1)
Monthly family income, minimum wages^‡^	
Up to 3	79 (20.9)
3.1-5	114 (30.2)
5.1-10	95 (25.1)
Above 10	86 (22.8)
Practice of physical activity	209 (55.3)
Frequency of physical activity	
Low frequency	122 (32.3)
Medium frequency	24 (6.4)
High frequency	63 (16.7)
Alcohol use	208 (55.0)
Smoking habit	32 (8.5)
Illicit drugs use	108 (28.6)
Others medication	129 (34.1)
Use of opioids	41 (10.8)

Results expressed as n (%). * Others: live with partner: 5.03%; widowed: 0.53%; separated: 1.59%; ^†^ did not respond: 0.5%; ^‡^ did not respond: 1.1%.

The consumption of methylphenidate at any time in life was reported by 37 students (37/378; 9.8%). Among these, 22 (22/37; 59%) declared its use for cognitive enhancement (22/378; 5.8%). The prevalence of recent methylphenidate use for cognitive enhancement was estimated at 2.4% (9/378). In general, 41% (9/22) of university students who used methylphenidate for cognitive enhancement, did it in the 4 week-period prior to the research.

Three (3/11; 27.3%) students who reported recent use of methylphenidate by self-medication had purchased the drug without a prescription. The purchase of the medication with no prescription was encouraged by friends in all cases. When methylphenidate was prescribed (n=8), the most prevalent diagnosis justifying its prescription was ADHD (5/8; 62.5%), and in 87.5% (7/8) of cases, the medication was prescribed by a psychiatrist. Recreational use (2/11; 9.1%) and reduction of daytime sleepiness (2/11; 9.1%) were also reported as justification for the off-label use of the medication.

Most university students reporting the use of methylphenidate for cognitive enhancement in the 4-week period prior to the survey were aged between 20 and 30 years (67%) and were female (56%) ( Table 3 ). Students living with their parents and who declared a monthly family income of more than 5.1 minimum wages also had greater involvement in cognitive enhancement practices with methylphenidate. Sedentarism, smoking habit and use of other medications, including opioids, appeared as variables associated with the practice of cognitive enhancement ( Table 3 ).

**Table 3:** Prevalence of use of methylphenidate for pharmacological cognitive enhancement in the 4-week period prior to the survey, according to socioeconomic and lifestyle variables

Variables	Recent use of methylphenidate		

	% (n=11)	Prevalence (95%CI)	Sample n (%) (n=378)
Age 20-30 years	66.7	2.11 (0.97-4.52)	285 (75.4)
Female	55.6	2.07 (0.89-4.76)	241 (63.8)
Undergraduate courses	55.6	1.92 (0.82-4.42)	260 (68.8)
Area of knowledge			
Graduate courses	44.4	3.39 (1.33-8.39)	118 (31.2)
Marital status single	88.9	2.77 (1.41-5.37)	289 (76.4)
Living with parents/siblings	66.7	3.28 (1.51-6.97)	183 (48.5)
Monthly family			
5.1 to 10 minimum wages	33.3	3.16 (1.08-8.88)	95 (25.1)
>10 minimum wages	33.3	3.49 (1.19-9.76)	86 (22.8)
No physical activity practice	66.7	3.55 (1.64-7.53)	169 (44.7)
Alcohol use	55.5	2.40 (1.03-5.50)	208 (55.0)
Non-smoker	88.9	2.31 (1.18-4.50)	346 (91.5)
No use of illicit drugs	55.5	1.85 (0.79-4.26)	270 (71.4)
No use of opioids	100	2.67 (1.41-5.00)	337 (89.2)
No use of medication	100	3.61 (1.91-6.73)	246 (65.9)

95%CI: 95% confidence interval.

**Figure 1:**
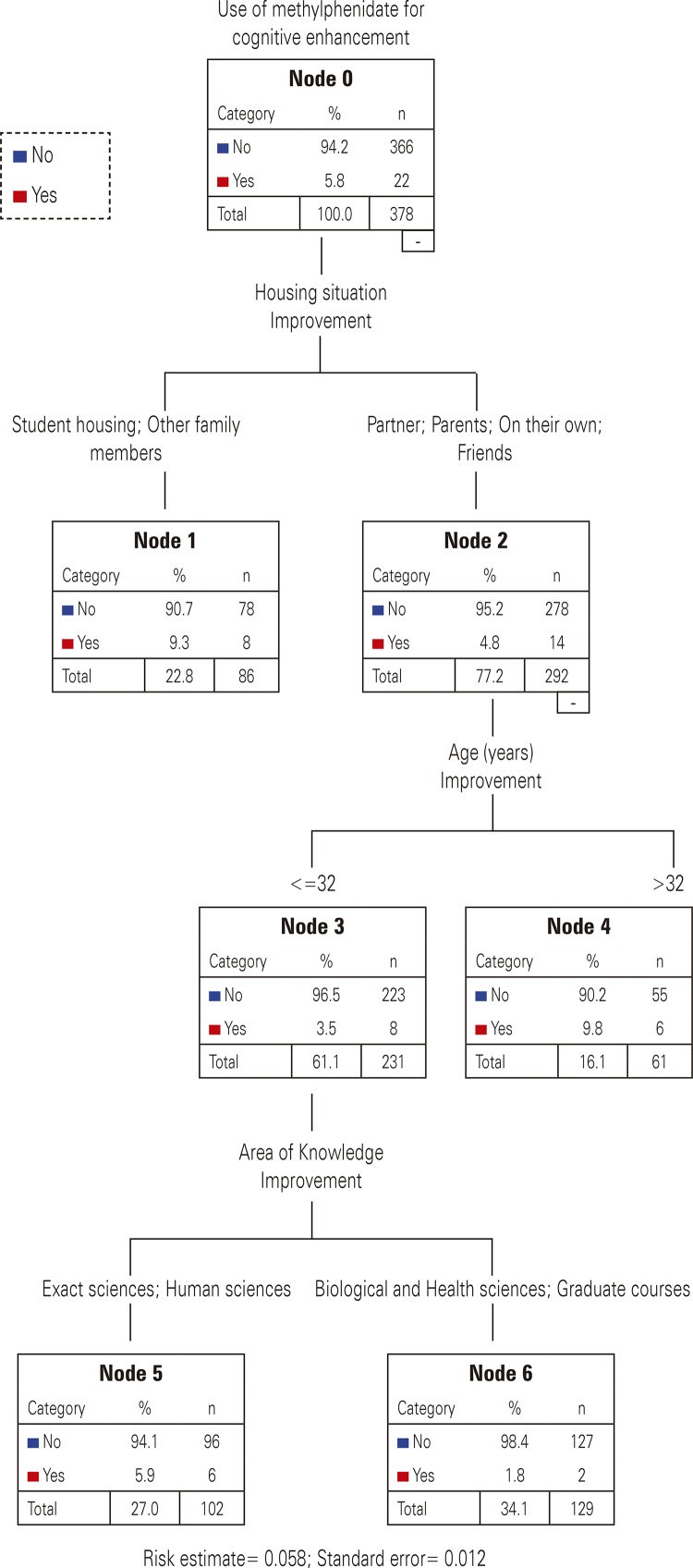
Decision tree for the classification of the use of metilphenidate

The group of university students who used methylphenidate for cognitive enhancement at any time in life is presented at the root node of the decision tree ( Figure 1 ). Living arrangement was the variable that mostly differentiated the students who used methylphenidate for cognitive enhancement, with a higher consumption trend also observed among students living with parents, with partner, with friends, or on their own, compared to those living in student housing or with other family members. The age of students was dichotomized as a result of the analysis, and those aged ≤32 years were identified as most likely to use methylphenidate for cognitive enhancement, when compared to students aged >32 years. Regarding areas of knowledge, Exact sciences and Human sciences students formed a group with greater probability of using methylphenidate for cognitive enhancement at any time in life. The decision tree method correctly classified 94.2% of methylphenidate use cases (risk estimate=0.058).

## DISCUSSION

The practice of pharmacological cognitive enhancement with the use of methylphenidate is present in the life of the university students evaluated. Considering the scientific literature in the area, this prevalence transcends the study population and extends, in a more or less accentuated way, to the Brazilian university population. Our results also allow us to infer that the prevalence of this practice among Brazilian university students equals or tends to equal the rates observed in the United States, where it is considered a public health problem.^9^ In that country, the practice of pharmacological cognitive enhancement was demonstrated in 6.9% of students from 39 universities^4^ and in 5.3% of university students aged 18 to 24 years.^5^

The improvement in academic performance is highlighted by about 60% of university students who engage in the illicit use of psychostimulants, including methylphenidate.^15^ This evidence motivates the monitoring of these young people, with the purpose of preventing abuse of these substances.^4^ The need to prevent this practice is enhanced by controversial evidence on the efficacy of methylphenidate for cognitive enhancement,^16^ and by the association of its use with serious cardiovascular risks, besides the exacerbation of preexisting psychotic or manic symptoms.^17 , 18^

In the present sample of university students, the recent use of methylphenidate for pharmacological cognitive enhancement was six times higher than consumption at any time in life. The characterization of the drug consumption in two periods (recent and at any time in life) allows to differentiate the use predominantly occurring in isolated events from the routine use, and also minimizes the participants’ response bias. This temporal characterization has not been systematically performed in similar studies, which may explain the finding of prevalence rates lower than that demonstrated in our study.^4 , 5^ Globally, the use of methylphenidate has significantly increased: between 2000 and 2013, the consumption of methylphenidate, in defined daily doses, increased from 500 million to approximately 2 billion and 400 million.^19^

Our study shows that approximately one-third of students who used methylphenidate purchased it without a prescription. The self-report of American university students shows a similar trend.^5^ Methylphenidate is classified as a psychotropic therapeutic product, and its sale is regulated by the *Agência Nacional de Vigilância Sanitária* (Anvisa) [Brazilian Health Regulatory Agency],^6 , 7^ with special prescription forms controlled by notification requirements. Although the *Sistema Nacional de Gerenciamento de Produtos Controlados* (SNGPC) [National System of Management of Controlled Products] represents a progress in the control of these sales, the report of acquisition of the drug without prescription is indicative of failures in the public policies for this control. These failures expose the population to health and legal risks, since the acquisition and sale of controlled products without a prescription outside of authorized venues are considered criminal offenses.^20^

The recommendation of friends was reported by the students who have acquired methylphenidate without prescription, showing a self-medication practice with methylphenidate similar to that observed in other studies with university students (>90%).^4^ In general, the practice of self-medication encouraged by family members and friends is quite common in the Brazilian population, and this practice, observed in university students with methylphenidate, is a reflection of this reality.^21^ In cases where methylphenidate has been prescribed, there are grounds for reflection. The efficacy and safety of methylphenidate in adults are not consistently established, and its prescription for this age group is not recommended, even for cases of ADHD or narcolepsy.^18^

The decision tree method used in our study has been used to complement or replace other multivariate analyses in many fields of research ( *e.g.* health cost analysis).^22^ The use of methylphenidate for cognitive enhancement is associated with the younger groups and with the Exact sciences and Human sciences group. The living arrangement analysis demonstrates that students living on their own are also more likely to use methylphenidate for cognitive enhancement,^23^ suggesting also that the support of close friends (parents, partners, and friends) is a protective factor for this practice. Age, social and behavioral variables are important determinants of non-medical use and abuse of drugs reserved for use under medical prescription,^24^ and the findings of this study can be useful for identifying target groups for future public policies and educational initiatives to prevent the inappropriate and abusive use of these medications.

Public policies should also consider the potential association between the consumption of methylphenidate for non-medical purposes, such as cognitive enhancement, and the consumption of opioid drugs and illicit drugs. In this sample, a high percentage of university students reported use of illicit drugs and opioids, and particularly in this age group, there is a potential risk of association between these behaviors.^25 , 26^

Potential limitations of the study refer to the inability of establishing a clear temporal relation between the use of methylphenidate and the exposure variables ( *e.g.* current housing), which is inherent to its cross-sectional design. In addition, due to the limited number of participants who had recently used methylphenidate for cognitive enhancement (4 weeks prior to the interview), it was not possible to determine the factors associated with this outcome. We also emphasize the fact that the study evaluated students from a single university, and it is impossible to generalize the results to the entire Brazilian university population. In spite of the limitations, our study pioneered in estimating the prevalence of the off-label use of methylphenidate for pharmacological cognitive enhancement among Brazilian university students, and should contribute to a broader discussion of a growing public health problem.

## CONCLUSION

The prevalence of methylphenidate use for pharmacological cognitive enhancement is sizable within a public health perspective, and it should be understood and addressed as such, both in the production of new knowledge and in the establishment of policies target to the problem. The highlighted evidence portrays the reality of this consumption in the university environment and deepen the discussion of the scenario, collaborating for the establishment of public policies to address it. Brazilian researchers should continue investigations in this field to provide a better assessment of the extent of the problem in our population and to prevent potential damages caused by the abusive and irrational consumption of methylphenidate.
