# Supplementary material for: Prevalence of and factors associated with the use of methylphenidate for cognitive enhancement among university students
Source: Einstein (Sao Paulo). 2019 Oct 17;18:eAO4745. doi: 10.31744/einstein_journal/2020AO4745 (PMC6896602; doi:10.31744/einstein_journal/2020AO4745)
Supplement: Translation

# Translation: Prevalência e fatores associados ao uso de metilfenidato para neuroaprimoramento farmacológico entre estudantes universitários

**Authors:** Raissa Carolina Fonseca Cândido, Edson Perini, Cristiane Menezes de Pádua, Daniela Rezende Junqueira

**Affiliations:** 1 Universidade Federal de Minas Gerais, Belo Horizonte, MG, Brasil.

**Keywords:** Metilfenidato, Cognição/efeito dos fármacos, Estimulantes do sistema nervoso central, Uso *off-label*, Estudantes, Prevalência

## Abstract

**Objetivo:**

Estimar a prevalência e os fatores associados ao uso de metilfenidato para neuroaprimoramento entre estudantes universitários.

**Métodos:**

Amostra aleatória simples de discentes da Universidade Federal de Minas Gerais (n=438), convidados a responder um questionário online sobre o consumo de metilfenidato. A coleta ocorreu de setembro de 2014 a janeiro de 2015. A amostra foi descrita em termos de proporções, médias e desvio padrão. A análise multivariada foi realizada utilizando o algoritmo *Classification and Regression Tree* para classificação dos casos de uso do metilfenidato para neuroaprimoramento em grupos, com base nas variáveis de exposição.

**Resultados:**

Dos 378 alunos incluídos, 5,8% (n=22) declararam ter feito uso de metilfenidato para neuroaprimoramento, sendo 41% (9/22) nas 4 semanas anteriores à pesquisa. A situação da moradia foi a variável mais associada ao uso de metilfenidato para neuroaprimoramento. Relataram o uso do metilfenidato para neuroaprimoramento e outros fins nas 4 semanas anteriores à pesquisa 11 estudantes, sendo que 27% não apresentaram prescrição médica para adquiri-lo.

**Conclusão:**

O uso de metilfenidato para neuroaprimoramento ocorre no meio acadêmico brasileiro e deve ser considerado sério problema de saúde pública, principalmente diante dos riscos de danos e efeitos adversos associados ao seu uso.

## INTRODUÇÃO

O metilfenidato é um medicamento estimulante do sistema nervoso central aprovado no Brasil para o tratamento do Transtorno do *deficit* de Atenção com Hiperatividade (TDAH) e narcolepsia. A utilização de substâncias psicoestimulantes, como o metilfenidato, para o neuroaprimoramento, tornou o *doping* uma prática que extrapolou o ambiente esportivo. Em muitos países, estudantes universitários têm utilizado medicamentos para melhorar o desempenho em avaliações e aumentar a capacidade de aprendizagem, criando um comércio paralelo de substâncias nos *campi* universitários.^1 , 2^ O uso do metilfenidato para neuroaprimoramento farmacológico se destaca nesse contexto, com prevalência estimada entre 5 e 7% entre universitários norte-americanos.^3 - 5^ A legislação brasileira classifica o metilfenidato na lista de psicotrópicos, junto de outras substâncias indutoras de dependência, como a metanfetamina (“ *ice* ”), sujeitando sua prescrição e seu uso a controle com notificação especial.^6 , 7^

As análises sobre o consumo de metilfenidato para fins não médicos abordam, em geral, questões relacionadas ao abuso e à dependência de drogas.^3^ Poucos estudos investigam a prevalência do uso de estimulantes para fins não médicos, analisando em profundidade o comportamento das pessoas que declaram esse consumo para neuroaprimoramento.^3 - 5^ Evidências científicas sobre a eficácia e os efeitos adversos do metilfenidato no neuroaprimoramento também são escassas.^8^ Não obstante, o uso abusivo de psicoestimulantes é considerado um problema de saúde pública nos Estados Unidos e no Reino Unido, onde pesquisas e debates em torno do assunto têm se fortalecido.^9 , 10^

No Brasil, um estudo das representações sociais sobre o uso de metilfenidato e o aprimoramento cognitivo farmacológico entre universitários evidenciou esta prática como realidade em nosso país.^11^ Considerando essa realidade e a carência de informações nesse contexto, torna-se relevante avaliar a prevalência do consumo de metilfenidato para neuroaprimoramento no meio universitário.

## OBJETIVO

Determinar a prevalência e os fatores associados ao uso de metilfenidato para fins de neuroaprimoramento de estudantes universitários brasileiros.

## MÉTODOS

### Delineamento do estudo

Estudo descritivo transversal realizado com os discentes da Universidade Federal de Minas Gerais (UFMG). Com sede na cidade de Belo Horizonte, a UFMG é uma das maiores universidades do país, com 47.088 alunos matriculados no ano de 2014, quando foi realizada a coleta de dados.^12^

### Participantes

Os estudantes foram selecionados a partir dos dados de registro na universidade. Para avaliar a representatividade da amostra resultante, os estudantes foram agrupados em estratos: três correspondentes às grandes áreas do conhecimento (exatas, humanas e biológicas/saúde) para alunos da Graduação, e outro, denominado Pós-Graduação, para alunos da Pós-Graduação e das Residências Médica e Multiprofissional. A área de conhecimento dos estudantes constava no cadastro na UFMG e também foi informada pelos participantes no questionário de pesquisa.

### Coleta de dados

As informações de registro de todos os alunos matriculados na UFMG foram organizadas em um banco de dados e, para cada aluno, foi designado um código. Uma amostra aleatória simples de discentes foi selecionada por meio de sorteio e recrutada por correio eletrônico. Os estudantes receberam três convites em intervalos de 15 dias. Os não respondentes foram substituídos de forma sistemática, de maneira a proporcionar a construção de uma amostragem aleatória simples sem reposição. Não respondentes após o terceiro contato, esgotadas as substituições, foram considerados perdas.

Os dados foram coletados por questionário eletrônico, organizado em três partes: variáveis sociodemográficas; dados sobre consumo do metilfenidato; e dados sobre hábitos de vida, incluindo prática de atividade física, hábito e fumar e uso de drogas ilícitas. A prática de atividade física foi analisada em quatro categorias: não pratica atividade física (referência); baixa frequência (30 a 45 minutos, três vezes por semana); média frequência (30 minutos, cinco vezes por semana, total de 2 horas e 30 minutos por semana); e alta frequência (mais de 2 horas e 30 minutos por semana). O uso do metilfenidato nas 4 semanas anteriores à pesquisa foi classificado como “recente”. O relato de uso de metilfenidato em período superior a 4 semanas correspondeu ao uso ocorrido em qualquer momento da vida.

Para a coleta e o armazenamento dos dados, utilizou-se a plataforma eletrônica SurveyMonkey^®^.

### Cálculo amostral

Considerando a prevalência de consumo de metilfenidato para neuroaprimoramento igual a 7%,^4^ erro amostral de 4%, nível de significância bicaudal de 5% e poder do estudo igual a 80%, adotando-se taxa de perdas de 25%, calculou-se amostra aleatória simples de 438 discentes matriculados nos cursos de graduação, pós-graduação e residência da UFMG (amostra calculada de 350, desconsiderando a taxa de perdas).

### Análise estatística

Os registros de respostas foram revisados manualmente para detecção de erros e inconsistências. As análises foram realizadas utilizando-se o software IBM (SPSS) *Statistics Base* , versão 22.0. Variáveis dicotômicas foram descritas utilizando-se proporções; variáveis contínuas foram descritas utilizando-se médias e desvios padrão. A análise da representatividade da amostra em relação à população com relação à área do conhecimento foi realizada utilizando-se o teste Z. A prevalência do consumo de metilfenidato foi calculada com intervalo de confiança de 95% (IC95%).

Para compreender o uso do metilfenidato para neuroaprimoramento com relação às variáveis de exposição, foi realizada análise multivariada pelo método de árvore de decisão. O método da árvore de decisão, utilizando o algoritmo *Classification and Regression Tree* (C&RT),^13^ foi utilizado para investigar variáveis preditoras do consumo do metilfenidato para o neuroaprimoramento como alternativa ao método da regressão logística binária, dado o limitado número de casos disponíveis para análise.

A árvore de decisão é uma técnica de partição recursiva utilizada para classificação e regressão dos dados em estratos menores, definindo subconjuntos (nós filhos) tão homogêneos quanto possível em relação ao nó raiz (população total contendo a variável dependente). O método C&RT maximiza a homogeneidade do nó, e a extensão de heterogeneidade dos casos é uma indicação de impureza. A medida de impureza empregada foi o coeficiente de Gini, que é baseado nas probabilidades quadradas de associação para cada categoria da variável dependente. O coeficiente de Gini varia entre zero e 1, e atinge seu valor mínimo quando todos os casos em um nó estão incluídos em uma mesma categoria (nó puro). A diferença entre o coeficiente de Gini para o nó raiz e a soma dos valores para os nós filhos, ponderada pela proporção de casos em cada nó filho, é apresentada na árvore como *improvement* . Esta medida representa a importância relativa do nó, avaliada pela diminuição na heterogeneidade do galho, quando comparado com o nó raiz (população total). O ajuste do modelo final foi avaliado por estimativa de risco, que identifica a proporção de casos que foram incorretamente classificados pelo modelo.^14^ Variáveis de exposição para o uso do medicamento foram incluídas simultaneamente no modelo, permanecendo no modelo final aquelas com maior valor do *improvement* .

O projeto foi aprovado pelo Comitê de Ética em Pesquisa da UFMG (parecer CEP 441.603), CAAE: 19606913.5.0000.5149.

## RESULTADOS

Foram incluídos 378 estudantes, sendo 69% da graduação. A amostra foi semelhante à população universitária para área do conhecimento, não obstante maior concentração de respondentes das áreas de Biológicas e Saúde e de Humanas ( Tabela 1 ).

**Tabela 1:** Distribuição dos discentes na amostra e na população universitária por área do conhecimento

Área do conhecimento	Amostra (n=378)	Alunos da Universidade (n=47.088)
Graduação		
Biológicas e Saúde	105 (27,8)	9.367 (20)*
Exatas	65 (17,2)	10.053 (21)
Humanas	90 (23,8)	14.453 (31)^†^
Pós-graduação, Residência Médica e Residência Multiprofissional	118 (31,2)	13.215 (28)

Resultados expressos como n (%). * p=0,0002 (teste Z);^†^p=0,0026 (teste Z).

A média de idade dos estudantes respondentes foi de 27,9 anos (desvio padrão de 8,2) e as mulheres representaram a maioria da amostra (64%). A maioria dos participantes declarou ser solteiro e morar com os pais, sendo a faixa de renda familiar mensal predominante de 3,1 a 5 salários mínimos (30,2%). Significante parcela dos estudantes não praticava atividade física habitualmente (45%). A maior parte dos entrevistados relatou fazer uso de bebidas alcóolicas (55%) e declarou não fumar (91,5%). O consumo de outros medicamentos, diferentes do metilfenidato, foi relatado por 34% dos estudantes. O uso de drogas ilícitas e de medicamentos opioides foi declarado por 39% da amostra, ocorrendo majoritariamente há 12 meses ou mais (40% e 44%, respectivamente) ( Tabela 2 ).

**Tabela 2:** Características demográficas e socioeconômicas, hábitos e estilo de vida dos discentes

Variáveis	
Sexo	
Feminino	241 (63,8)
Idade, anos	
≤19	5 (1,3)
20-30	285 (75,4)
>30	88 (23,3)
Estado civil	
Solteiro(a)	289 (76,4)
Casado(a)	62 (16,4)
Outros*	27 (7,2)
Moradia^†^	
Com os pais	183 (48,5)
Com companheiro	73 (19,4)
Com outros familiares	35 (9,3)
Com amigos	18 (4,8)
Em república	45 (11,4)
Sozinho	23 (6,1)
Renda familiar mensal, salários mínimos^‡^	
Até 3	79 (20,9)
3,1-5	114 (30,2)
5,1-10	95 (25,1)
Acima de 10	86 (22,8)
Prática de atividade física	209 (55,3)
Frequência de atividade física	
Baixa frequência	122 (32,3)
Média frequência	24 (6,4)
Alta frequência	63 (16,7)
Uso de bebida alcóolica	208 (55,0)
Hábito de fumar	32 (8,5)
Uso de drogas ilícitas	108 (28,6)
Uso de outros medicamentos	129 (34,1)
Uso de opioides	41 (10,8)

Resultados expressos como n (%). * Outros: mora com companheiro: 5,03%; viúvo: 0,53%; separado: 1,59%;^†^não responderam: 0,5%;^‡^não responderam: 1,1%.

O consumo de metilfenidato, independente do momento da vida, foi relatado por 37 estudantes (37/378; 9,8%). Dentre estes, 22 (22/37; 59%) declararam ter feito seu uso para neuroaprimoramento (22/378; 5,8%). A prevalência de consumo recente de metilfenidato para neuroaprimoramento foi estimada em 2,4% (9/378). Em geral, 41% (9/22) dos universitários que usaram metilfenidato para neuroaprimoramento, realizou o uso nas 4 semanas anteriores à pesquisa.

Três (3/11; 27,3%) dos estudantes que fizeram uso recente do metilfenidato por automedicação adquiriram o medicamento sem prescrição médica. A aquisição do medicamento sem prescrição médica foi incentivada por indicação de amigos em todos os casos. Nos casos em que o metilfenidato foi prescrito (n=8), o diagnóstico mais prevalente justificando sua prescrição foi o TDAH (5/8; 62,5%), sendo que, em 87,5% (7/8) dos casos, o medicamento foi prescrito por médico psiquiatra. O uso recreativo (2/11; 9,1%) e a diminuição do sono diurno (2/11; 9,1%) também foram relatados como justificativa para o uso *off-label* do medicamento.

Os universitários que relataram o consumo de metilfenidato para neuroaprimoramento nas 4 semanas anteriores à pesquisa possuíam, em sua maioria, idade entre 20 e 30 anos (67%) e eram do sexo feminino (56%) ( Tabela 3 ). Estudantes morando com os pais e que declararam renda familiar mensal superior a 5,1 salários mínimos também apresentaram maior envolvimento em práticas de neuroaprimoramento com o metilfenidato. Sedentarismo, hábito de fumar e uso de outros medicamentos, dentre eles os opioides, apareceram como variáveis associadas à prática do neuroaprimoramento ( Tabela 3 ).

**Tabela 3:** Prevalência do uso de metilfenidato para neuroaprimoramento farmacológicos nas 4 semanas anteriores à pesquisa, conforme variáveis socioeconômicas e de estilo de vida

Variáveis	Uso recente de metilfenidato		

	% (n=11)	Prevalência (IC95%)	Amostra n (%) (n=378)
Idade 20-30 anos	66,7	2,11 (0,97-4,52)	285 (75,4)
Sexo feminino	55,6	2,07 (0,89-4,76)	241 (63,8)
Graduação	55,6	1,92 (0,82-4,42)	260 (68,8)
Área do conhecimento			
Pós-Graduação	44,4	3,39 (1,33-8,39)	118 (31,2)
Estado civil solteiro	88,9	2,77 (1,41-5,37)	289 (76,4)
Moradia com os pais/irmãos	66,7	3,28 (1,51-6,97)	183(48,5)
Renda familiar mensal			
5,1 a 10 salários mínimos	33,3	3,16 (1,08-8,88)	95 (25,1)
>10 salários mínimos	33,3	3,49 (1,19-9,76)	86 (22,8)
Não pratica de atividade física	66,7	3,55 (1,64-7,53)	169 (44,7)
Faz uso de bebida alcóolica	55,5	2,40 (1,03-5,50)	208 (55,0)
Não tem hábito de fumar	88,9	2,31 (1,18-4,50)	346 (91,5)
Não faz uso de drogas ilícitas	55,5	1,85 (0,79-4,26)	270 (71,4)
Não faz uso de opioides	100	2,67 (1,41-5,00)	337 (89,2)
Não faz uso de medicamentos	100	3,61 (1,91-6,73)	246 (65,9)

IC95%: intervalo de confiança de 95%.

O grupo de estudantes que fez uso do metilfenidato para neuroaprimoramento em algum momento da vida é apresentado no nó raiz da árvore de decisão ( Figura 1 ). A situação de moradia foi a variável que melhor discriminou os estudantes que utilizaram metilfenidato para neuroaprimoramento, com maior tendência de consumo igualmente observada entre os estudantes que moravam com pais, companheiro, amigos ou sozinhos em comparação àqueles que moravam em repúblicas ou com outros familiares. A idade dos estudantes foi dicotomizada como resultado da análise, e aqueles com idade ≤32 anos foram identificados como mais propensos ao uso do metilfenidato para neuroaprimoramento, quando comparados com estudantes com idade >32 anos. Com relação às áreas do conhecimento, os estudantes de exatas e humanas formaram um grupo mais propenso a utilizar o metilfenidato para neuroaprimoramento, independente do momento da vida. O método da árvore de decisão classificou corretamente 94,2% dos casos de uso de metilfenidato (estimativa de risco =0,058).

**Figura 1:** Árvore de decisão para classificação do uso do metilfenidato

## DISCUSSÃO

A prática de neuroaprimoramento farmacológico com uso de metilfenidato está presente na vida dos estudantes universitários avaliados. Considerando a literatura científica na área, essa prevalência transcende a população do estudo e se estende, de forma mais ou menos acentuada, à população universitária brasileira. Nossos resultados também permitem inferir que a prevalência dessa prática entre universitários brasileiros se equipara ou tende aos índices conhecidos nos Estados Unidos, onde é considerada um problema de saúde pública.^9^ Naquele país, a prática de neuroaprimoramento farmacológico foi demonstrada em 6,9% dos estudantes de 39 universidades^4^ e em 5,3% dos universitários com idade entre 18 e 24 anos.^5^

A busca por melhoria do desempenho acadêmico é apontada por cerca de 60% dos estudantes universitários que realizam o uso ilícito de psicoestimulantes, incluindo o metilfenidato.^15^ Essas evidências motivam o acompanhamento desses jovens, com o propósito de prevenir o abuso dessas substâncias.^4^ A necessidade de prevenção dessa prática é reforçada por evidências controversas sobre a eficácia do uso de metilfenidato para neuroaprimoramento,^16^ e pelas relações entre seu uso e riscos cardiovasculares graves, além da exacerbação de sintomas psicóticos ou maníacos preexistentes.^17 , 18^

Na presente amostra de estudantes universitários, o consumo recente de metilfenidato para neuroaprimoramento farmacológico foi seis vezes superior ao consumo realizado em qualquer momento da vida. A caracterização do consumo do medicamento em dois períodos (recente e em qualquer momento da vida) permite diferenciar o uso ocorrendo predominantemente em eventos isolados do uso rotineiro, além de minimizar o viés da resposta dos participantes. Essa caracterização temporal não tem sido realizada de forma sistemática em estudos semelhantes, o que pode explicar a descrição de prevalências inferiores à demonstrada em nosso estudo.^4 , 5^ Globalmente, o uso do metilfenidato tem aumentado significativamente: entre os anos 2000 e 2013, o consumo de metilfenidato, em doses diárias definidas, aumentou de 500 milhões para aproximadamente 2 bilhões e 400 milhões.^19^

Nosso estudo evidencia que aproximadamente um terço dos estudantes que utilizaram o metilfenidato o adquiriu sem prescrição médica. O relato espontâneo de estudantes universitários norte-americanos aponta tendência semelhante.^5^ O metilfenidato é classificado entre os medicamentos psicotrópicos, cuja comercialização é regulamentada pela Agência Nacional de Vigilância Sanitária (Anvisa),^6 , 7^ controlada por notificação de receita especial. Embora o Sistema Nacional de Gerenciamento de Produtos Controlados (SNGPC) represente um progresso no controle dessa comercialização, o relato da aquisição sem prescrição é indicativo de falhas nas políticas públicas direcionadas a esse controle. Essas falhas expõem a população a riscos à saúde e a riscos legais, uma vez que a aquisição e a comercialização de produtos controlados sem prescrição e fora de estabelecimentos autorizados são tipificadas como delitos penais.^20^

A indicação de amigos foi declarada pelos estudantes que adquiriram o metilfenidato sem prescrição médica, evidenciando prática de automedicação com metilfenidato semelhante à observada em outros estudos com universitários (>90%).^4^ De maneira geral, a automedicação estimulada por familiares e amigos é bastante comum na população brasileira, e tal prática, observada nos estudantes universitários com o metilfenidato, é reflexo dessa realidade.^21^ Nos casos em que o metilfenidato foi prescrito, também há motivos para reflexão. A eficácia e a segurança do uso do metilfenidato em adultos não estão estabelecidas de forma consistente, e sua prescrição para essa faixa etária não é recomendada, mesmo para os casos de TDAH ou narcolepsia.^18^

O método da árvore de decisão utilizado em nosso estudo tem sido empregado para complementar ou substituir outras análises multivariadas em muitos campos de pesquisa (por exemplo, na análise de custos em saúde).^22^ O uso do metilfenidato para aprimoramento está associado aos grupos mais jovens e das áreas exatas e humanas. A análise da situação de moradia demonstra que estudantes que moram sozinhos também são mais propensos ao uso do metilfenidato para aprimoramento,^23^ ao mesmo tempo em que sugere o apoio de entes próximos (pais, companheiros e amigos) como fator protetor para essa prática. Idade, variáveis sociais e comportamentais são importantes determinantes do uso não médico e abusivo de medicamentos reservados para utilização sob prescrição médica,^24^ e as observações desse estudo podem ser úteis para a identificação de grupos-alvo para futuras políticas públicas e ações educativas, para prevenir o uso inadequado e abusivo do medicamento.

Políticas públicas devem considerar também a potencial associação entre motivações não médicas para o consumo do metilfenidato, como para fins de neuroaprimoramento, e o consumo de medicamentos opioides e drogas ilícitas. Na amostra estudada, foi elevado o percentual de universitários que declararam uso de drogas ilícitas e opioides, e, particularmente, nessa faixa etária, existe potencial associação de risco entre esses comportamentos.^25 , 26^

Potenciais limitações do estudo referem-se à impossibilidade de se estabelecer uma relação temporal clara entre o uso do metilfenidato e as variáveis de exposição (exemplo: moradia atual), inerentes ao delineamento transversal. Ademais, devido ao número restrito de participantes que fizeram uso recente do metilfenidato para neuroaprimoramento (4 semanas anteriores à entrevista), não foi possível a determinação dos fatores associados a esse desfecho. Ressalta-se também o fato de o estudo avaliar estudantes de uma única universidade, impossibilitando a generalização dos resultados para toda a população universitária brasileira. Apesar das limitações apontadas, nosso estudo foi pioneiro em estimar a prevalência do uso ( *off label* ) do metilfenidato para o neuroaprimoramento farmacológico entre universitários brasileiros, e deve contribuir para a ampliação da discussão de um problema de saúde pública crescente.

## CONCLUSÃO

A prevalência de uso do metilfenidato para neuroaprimoramento farmacológico é considerável dentro de uma perspectiva de saúde pública, devendo ser compreendida e abordada como tal, seja na produção de novos conhecimentos, seja no estabelecimento de políticas de controle do problema. As evidências destacadas retratam a realidade desse consumo no meio universitário e aprofundam a discussão do problema, colaborando para a construção de políticas públicas que possam solucioná-lo. Investigações devem continuar sendo realizadas no Brasil para que possamos dimensionar melhor o problema em nossa população e prevenir os danos que o consumo abusivo e irracional do metilfenidato pode ocasionar.
